# Effectiveness of an oral care tablet containing kiwifruit powder in reducing oral bacteria in tongue coating: A crossover trial

**DOI:** 10.1002/cre2.262

**Published:** 2019-12-03

**Authors:** Yuki Matsumura, Daisuke Hinode, Makoto Fukui, Masami Yoshioka, Hiroki Asakuma, Hiroshi Takii

**Affiliations:** ^1^ Department of Hygiene and Oral Health Science Tokushima University Graduate School of Biomedical Sciences Tokushima Japan; ^2^ Faculty of Health and Welfare Tokushima Bunri University Tokushima Japan; ^3^ Institute of Health Sciences, Ezaki Glico Co., Ltd. Osaka Japan

**Keywords:** *Fusobacterium nucleatum*, kiwifruit powder, oral care tablet, oral malodor, tongue‐coating bacteria

## Abstract

**Objectives:**

The aim of this study was to investigate the effect of an oral care tablet containing kiwifruit powder on oral bacteria in tongue coating compared with tongue brushing.

**Material and methods:**

Thirty‐two healthy, young adults were enrolled, and a crossover clinical trial was conducted. The volatile sulfur compound (VSC) concentration, Winkel tongue‐coating index (WTCI), and the number of total bacteria in addition to *Fusobacterium nucleatum* in tongue coating were measured. We instructed subjects to remove tongue coating by tongue brush for Intervention I, to keep the oral care tablet containing kiwifruit powder on the tongue dorsum and to let it dissolve naturally for Intervention II, and three oral care tablets 1 day before the measurement for Intervention III.

**Results:**

There were significant differences in terms of the level of H_2_S, VSC, and WTCI at Intervention I and all evaluation values at Intervention II. There were significant differences in terms of the level of H_2_S, VSC, WTCI, the number of total bacteria, and *F. nucleatum* at Intervention III. The value of WTCI, the number of bacteria, and *F. nucleatum* decreased significantly after taking the oral care tablets than after tongue brushing. When compared with Interventions I and III, Intervention III showed the effective results; there were significant differences in the number of total bacteria and *F. nucleatum* between tongue brushing and taking tablets.

**Conclusions:**

These results suggested that the oral care tablet containing kiwifruit powder could be effective in reducing total bacteria and *F. nucleatum* in tongue coating when compared with tongue brushing.

## 1 INTRODUCTION

Oral malodor is one of the concerns among a large number of people in recent years. In the report of the Japanese Survey of Dental Diseases,(Report on the Survey of Dental Diseases, 2016) the percentage of people with concern about their bad breath was 9.6%. It reported that oral malodor was caused mainly by volatile sulfur compounds (VSCs) in mouth air, and these include hydrogen sulfide (H_2_S), methyl mercaptan (CH_3_SH), and dimethyl sulfide [(CH_3_)_2_S].(Tonzetich, 1971) Moreover, oral bacteria related to periodontal disease are capable of producing large amounts of VSCs.(Nakano, Yoshimura, & Koga, 2002; Shibuya, 2001) Among them, *Fusobacterium nucleatum* is known to the periodontal pathogen implicated in oral malodor due to different substances such as H_2_S and CH_3_SH results from bacterial metabolic activity.(Claesson, Edlund, Persson, & Carlsson, 1990; Nakano et al., 2002)

Halitosis is classified as pathological and physiological one.(Murata, Yamaga, Iida, & Miyazaki, 2002) Tongue coating causes physiological halitosis and pathological halitosis.(Yaegaki & Sanada, 1992a) The tongue dorsum is the largest surface in the mouth, and its papillary structure is complicated and highly colonized by bacteria. (Gordon & Gibbons, 1966; Kojima, 1985; Nakano et al., 2002) Tongue coating is a kind of biofilm formed on the dorsum and consists of epithelial cell debris, blood cells, and food debris in addition to oral bacteria that metabolize these substrates. Thus, tongue coating is a rich source of VSCs because of the large bacterial population. (Nakano et al., 2002) It has also been reported that approximately 60% of VSCs originate from the tongue surface in patients with periodontitis.(Yaegaki & Sanada, 1992b) These findings suggest that assessment of tongue coating deposition may be a good indicator of oral malodor. However, we had previously reported that 70% of subjects with highly accumulated tongue coating did not recognize their tongue coating and that half of the subjects had no habit of daily tongue cleaning.(Amou, Hinode, Yoshioka, & Grenier, 2014) Even healthy people, as well as patients complaining of oral malodor, should recognize if they have tongue coating and remove the accumulated coating effectively.

Mechanical cleaning using tongue brush is effective in removing tongue coating.(Slot, De Geest, van der Weijden, & Quirynen, 2015; Yaegaki, Coil, Kamemizu, & Miyazaki, 2002) However, there are few reports on chemical cleaning towards tongue coating. Yoshimatsu et al. conducted a study using an oral care tablet containing cysteine protease (actinidin) from kiwifruit and reported that the tablets were effective for chemical cleaning (Yoshimatsu et al., 2006) and suppressing VSC. (Nohno, Yamaga, Kaneko, & Miyazaki, 2012; Yoshimatsu et al., 2007) Protease should be effective in reducing and removing protein on the tongue dorsum (Tonzetich, Coil, & Ng, 1991; Tonzetich, Eigen, King, & Weiss, 1967; Tonzetich & McBride, 1981) because the main component of tongue coating is protein. However, few studies showed the effect of actinidin from kiwifruit powder on oral bacteria.

We had the opportunity to obtain the oral care tablet containing kiwifruit powder, which has already been approved in Japan. The aim of this study was to investigate the effect of an oral care tablet containing kiwifruit powder on oral bacteria in tongue coating and VSC concentration.

## 2 METHODS

### 2.1 Subjects and oral care tablet

**Figure 1:**
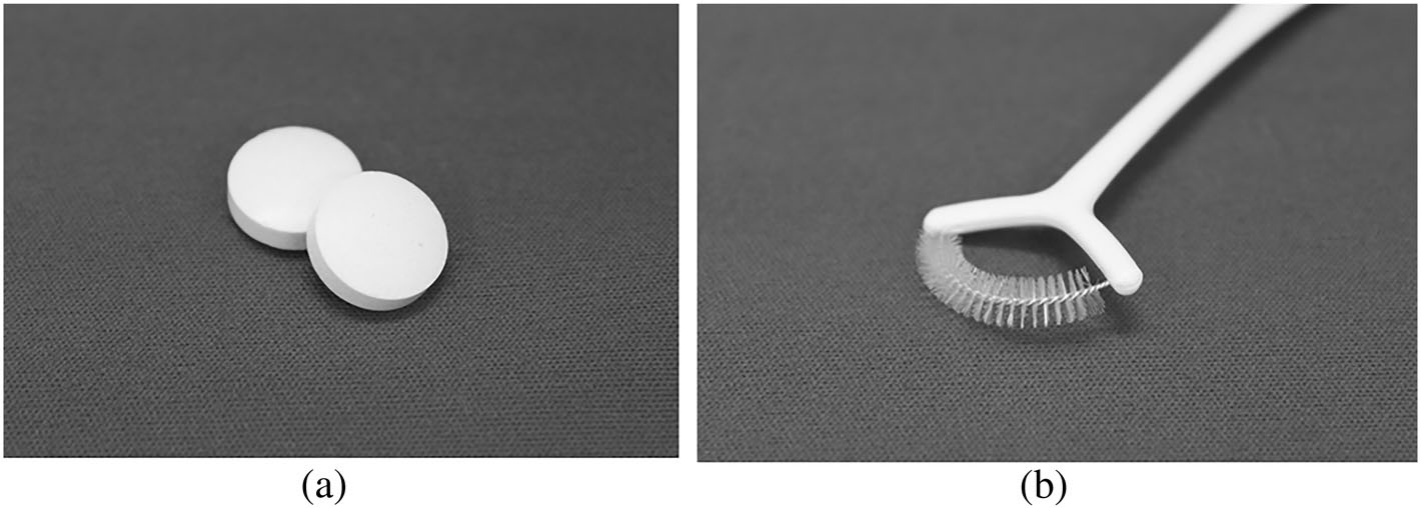
(a) Oral care tablet; (b) Tongue brush

Thirty‐two healthy students (5 males and 27 females; mean age 21.5 ± 2.1 years), who belonged to Tokushima University were enrolled in this study. Before enrollment, the subjects were informed about the methods and objectives of the study, and they provided a written informed consent. Participants were dentulous men and women, 18 years of age or older. Current smokers, pregnant women, and participants who had received an antibiotic treatment within the previous 2 weeks or who showed allergy against kiwifruit were excluded from the study. Oral care tablet (Figure 1a) was provided by Ezaki Glico Co., Ltd. (Osaka, Japan). Table 1 shows the composition of the tablet.

**Table 1:** Composition of oral care tablet

Reduced palatinose
Erythritol
Malted rice extract powder
Kiwifruit powder
Sorbitol
Food flavor
Acidulant
Emulsifier
Silica particulate
Calcium stearate
Sweetener
Food color
Tea extract

### 2.2 Study design

The sample size was obtained as follows: the data of the number of oral bacteria after using the oral care tablet and that of tongue brush were obtained from the results of five participants in our preliminary study. The primary variable was that the number of bacteria (log [cells per milliliter]), and the sample size was based on a two‐tailed *t* test with a significant difference level of 0.05, a power level of 0.90, and with an anticipated effect size *d* = difference of means/standard deviation = 1.19. The required sample size was 16 in each group for a total of 32.

**Figure 2:**
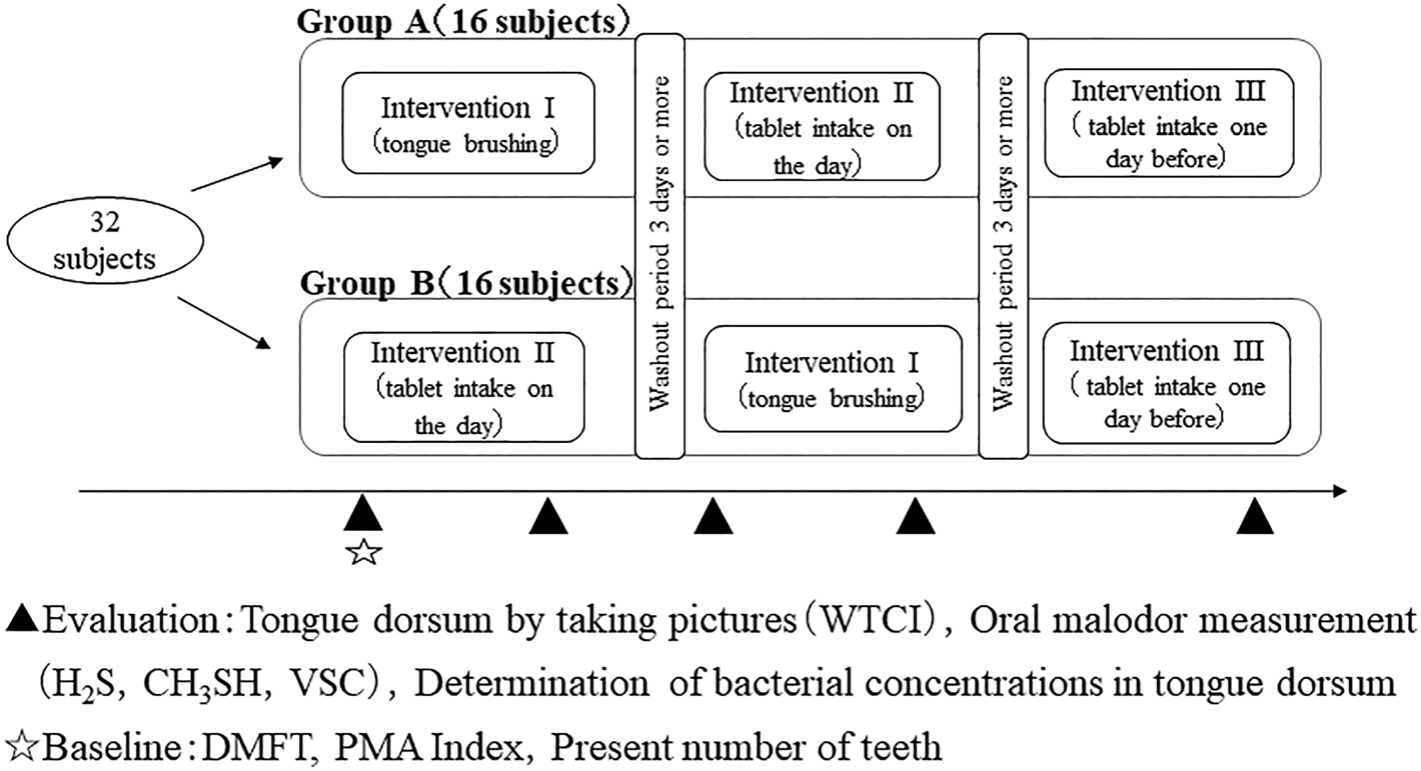
Outline of the crossover study

Figure 2 shows the outline of the crossover trial for 32 subjects in this study. The crossover clinical trial was conducted between Group A (16 subjects) and Group B (16 subjects). Group A performed in the order of Intervention I, Intervention II, and then Intervention III, whereas Group B performed in the order of Intervention II, Intervention I, and then Intervention III. These crossover studies had a washout period of 3 days or more. Closed triangle in Figure 2 shows the time of evaluation in this intervention study. Prior to the assignment for these assessments, each subject was asked to refrain from eating, drinking, and tooth brushing during the periods from waking up to the end of the trial and tongue cleaning within the past 3 days.

**Figure 3:**
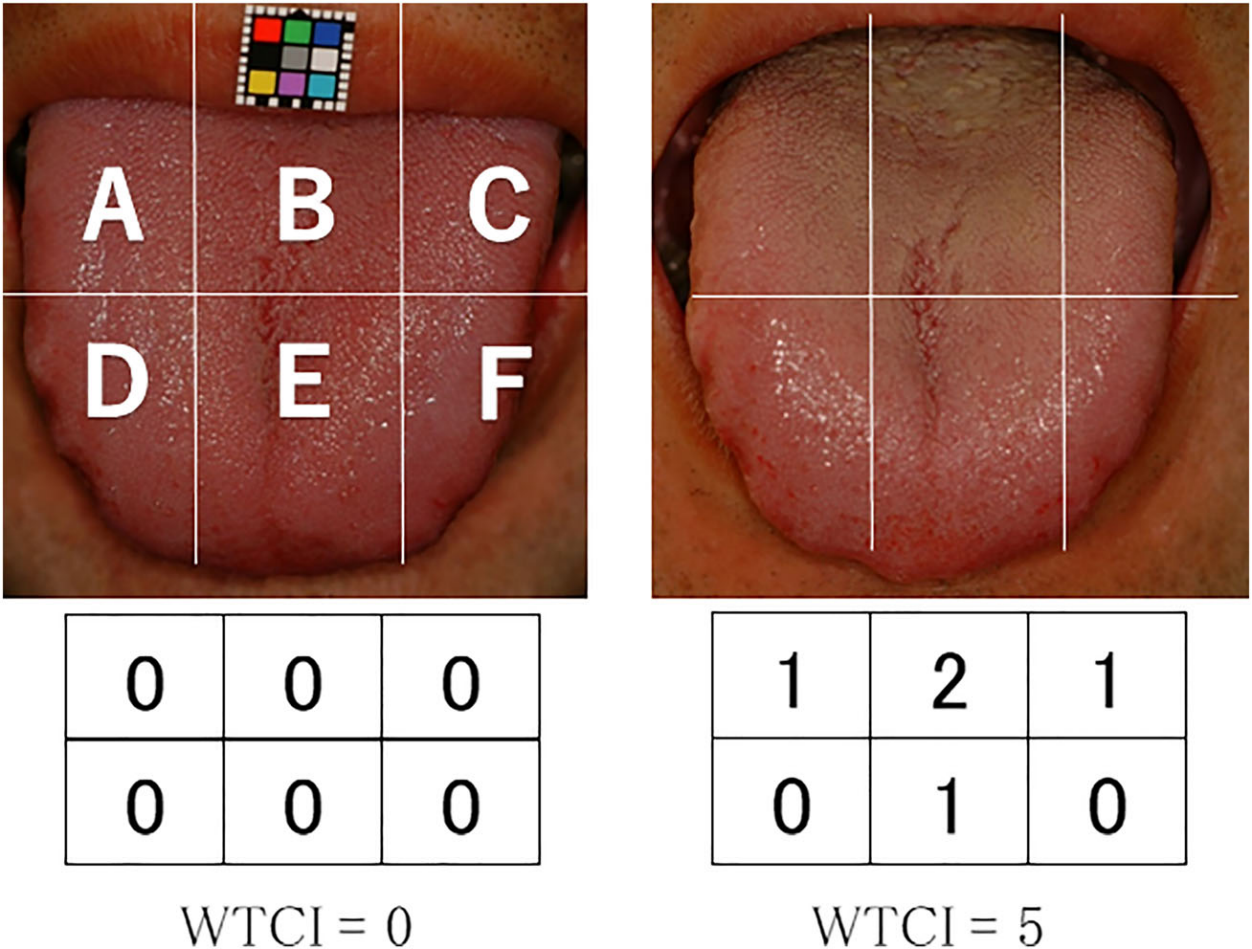
Score of Winkel tongue‐coating index and evaluation example

For Intervention I, the protocol of the clinical trial is as follows: (a) pictures of the dorsum were taken using a digital camera for the measurement of Winkel tongue‐coating index (WTCI); (b) VSC concentration was measured with Oral Chroma™ (Nissha FIS Co. , Ltd., Osaka, Japan); and (c) the number of total bacteria in tongue coating was measured by Bacteria Counter™ (Panasonic Co., Ltd., Osaka, Japan) on the day of the experiment. We instructed subjects about tongue cleaning by scrubbing 10 times from back to front with tongue brush in Figure 1b then washing with 10‐ml water. The subjects repeated the above procedure two times. After the intervention of 1 hr, Steps a–c were repeated. Regarding the protocol of Intervention II, we also carried out Steps a–c, then instructed subjects to take an oral care tablet (Figure 3) and to keep it on the tongue dorsum to let it dissolve naturally. Each participant digested two tablets. After an intervention of 1 hr, Steps a–c were repeated.

Regarding the protocol of Intervention III, we instructed the subjects to take one tablet three times a day after every meal before the measurement, and Steps a–c were performed on the next day. The data obtained from Intervention III were compared with that of the baseline. It is necessary to have a washout period to interrupt tongue brush for 3 days before each intervention study. We anticipated that it took 1 week to complete the examination, and we set the examination period for more than 3 weeks.

### 2.3 Oral assessment

#### 2.3.1 Evaluation of tongue coating

The accumulation of tongue coating was assessed by visual examination on the basis of WTCI. Figure 3a shows tongue dorsum of six divisions (*A* to *F*): 0 = *not visible*, 1 = *thin coating*, and 2 = *thick coating.* The scores were assigned by comparison with standard color photographs of tongue coating by one dentist and two dental hygienists as a single blind. The score of WCTI by the evaluation in Figure 3a,b were 0 and 5, respectively.

#### 2.3.2 Assessment of oral malodor

VSC was measured by Oral Chroma™ according to the manufacturer's instructions. The total amount of hydrogen sulfide (H_2_S), methyl mercaptan (CH_3_SH), and dimethyl sulfide [(CH_3_)_2_S] was defined as “VSC.” Before intervention after waking up, food intake was prohibited for participants.

#### 2.3.3 Evaluation of other items

The present number of teeth, decayed, missing and filled teeth index, and papillary, marginal and attached gingiva index were also evaluated to characterize the subjects.

### 2.4 Measurement of the number of total bacteria

The dielectrophoretic impedance measurement apparatus for quantification of bacteria (Bacterial Counter™) was used to assess tongue‐coating bacteria according to the manufacturer's instructions. Each tongue‐coating sample was collected using a sterile 5‐mm diameter cotton stick by swabbing the tongue dorsum three times from back to front (approximately 2‐cm long swabbing motions). Samples were suspended in 5 ml of distilled water in a disposable cup, and bacterial quantification with Bacterial Counter™ was performed. After that, the samples were dispensed into vials and kept at −80°C until used for specific bacterial quantification by real‐time polymerase chain reaction (PCR).

### 2.5 Determination of bacterial concentrations by real‐time PCR

Tongue coating samples were also used to quantify periodontopathogenic bacteria (*F. nucleatum*) by quantitative PCR as previously reported by Moriyama et al. (Moriyama et al., 2018) with slight modifications. The MiniOpticon system (Bio‐Rad Laboratories, Hercules, CA, USA) with SYBR Green I dye was used for the quantitative PCR analysis. One hundred eighty microliter of InstaGene Matrix (Bio‐Rad Laboratories) was added to 20 μl of each tongue coating sample. The mixtures were incubated at 56°C for 30 min, vortexed for 30 s, incubated at 100°C for 8 min, and then stored at −20°C until used for the quantitative PCR analysis. Before analysis, the mixtures were thawed and centrifuged at 10,000 g for 10 min at 4°C. The supernatant of the samples was used for DNA template and was added (2 μl) to the PCR reaction mixture (18 μl) made of 10 μl of SsoFast™ EvaGreen® Supermix (Bio‐Rad Laboratories), 0.04 μl of 100 μM of primers (forward, reverse), and 7.92 μl of diethylpyrocarbonate‐treated water. The liquid mixtures were heat treated as follows: initial denaturation step (3 min at 95°C), followed by denaturation (5 s at 95°C), annealing (10 s at 60°C), and extension (10 s at 60°C). The number of cycles for *F. nucleatum* was 38. The primers used for the quantitative PCR have been previously described.(Yokoyama et al., 2008) A standard curve was generated on the basis of the known number of *F. nucleatum* ATCC 23726. Ten‐fold serial dilutions of bacterial standards of *F. nucleatum* were prepared, and each extracted DNA was used. The concentrations of *F. nucleatum* in tongue coating samples were calculated from the number of copies of the target sequence.

### 2.6 Statistical analyses

Data were analyzed using the software IBM SPSS Statistics Ver. 23 (SPSS Japan Inc., Tokyo). The difference between the two groups with baseline was assessed using the Mann–Whitney *U* test or Student's *t* test. For the analysis of the carryover effect and the period effect in this crossover clinical trial, each chronological sequence data were prepared and then assessed using the Mann–Whitney *U* test or Student's *t* test. The effect of each intervention study was analyzed by the Wilcoxon test or paired *t* test. Comparison of the effect of intervention study was analyzed by the Mann–Whitney *U* test or Student's *t* test.

### 2.7 Ethics

The ethics committee of Tokushima University Hospital approved this study (Protocol Approval Number 2923). The method and objectives of this study were explained to the subjects who provided written informed consent before their participation in the study.

## 3 RESULTS

### 3.1 Comparison of the item at baseline, the carryover effect, and the period effect

The mean ± standard deviation of decayed, missing, and filled teeth index, papillary marginal attachment index, and the number of present teeth in subjects were 2.7 ± 3.9, 0.2 ± 0.4 and 28.1 ± 1.9, respectively. No significant difference of items at baseline between Groups A and B was observed (Table 2). It is necessary to consider the carryover effect, which is defined as the lingering effect of the treatment of the previous study period on the current study period.(Wang, Cong, ChenT, & Zhang, 2019) Also, it is necessary to consider the period effect, which represents a systematic difference between different periods in the outcome for evaluating treatment. There was no significant difference in all items observed regarding the carryover effect and the period effect, as shown in Table 3. These were not influenced in this crossover study.

**Table 2:** Comparison of items at baseline

		All subjects(32)					Group A(16)					Group B (16)				
		Males: females (5:27)					Males: females (2:14)					Males: females (3:13)				
Items	Average value	Standard division	Median value	Percentile		Average value	Standard division	Median value	Percentile		Average value	Standard division	Median value	Percentile		*p* value
				25	75				25	75				25	75	
Age	21.5	2.1	21.0	20.0	22.8	21.2	1.5	21.0	20.0	22.0	21.8	2.6	21.0	20.0	23.0	0.408^a^
Total bacteria (log_10_[cell/ml])	7.3	0.3	7.3	7.0	7.6	7.2	0.3	7.3	7.0	7.5	7.4	0.3	7.4	7.1	7.6	0.154^a^
H_2_S (ppb)	78.2	97.2	27.0	4.0	160.0	104.6	115.0	51.5	3.3	205.8	51.8	69.4	21.5	4.5	72.3	0.423^b^
CH_3_SH (ppb)	17.8	30.2	0.0	0.0	32.0	21.4	28.4	0.0	0.0	50.3	14.3	32.3	0.0	0.0	3.0	0.616^b^
VSC (ppb)	100.2	127.5	27.0	4.0	213.3	132.8	144.9	82.0	3.3	251.3	67.6	101.8	21.5	5.8	74.5	0.515^b^
WTCI	5.8	2.2	6.0	4.0	7.0	5.7	2.1	6.0	4.0	7.0	6.0	2.4	6.0	4.5	7.0	0.564^b^
DMFT	2.7	3.9	1.0	0.0	4.0	2.0	3.0	0.0	0.0	3.8	3.4	4.6	1.5	0.0	7.0	0.402^b^
PMA index	0.2	0.4	0.0	0.0	0.0	0.1	0.3	0.0	0.0	0.0	0.2	0.5	0.0	0.0	0.0	0.985^b^
The number of present teeth	28.1	1.9	28.0	28.0	29.5	28.3	2.0	28.0	28.0	29.5	28.0	1.8	28.0	27.3	29.5	0.780^b^

Abbreviations: DMFT, decayed, missing, and filled teeth index; PMA, papillary marginal attachment; ppb, parts per billion; VSC, volatile sulfur compound; WTCI, Winkel tongue‐coating index.

a Student's *t* test.

b Mann–Whitney *U* test

**Table 3:** Carryover effect and period effect regarding the crossover study of Interventions I and II

	H_2_S^a^	CH_3_SH^a^	VSC^a^	WTCI^a^	Total bacteria^b^
Carryover effect	0.445	0.361	0.423	0.184	0.509
Period effect	0.341	0.224	0.254	1.000	0.415

*Note.* The value means *p* value by the statistical analysis.

Abbreviations: VSC, volatile sulfur compound; WTCI, Winkel tongue‐coating index.

a Mann–Whitney *U* test.

b Student's *t* test.

### 3.2 Comparison of the effect of each intervention study

**Figure 4:**
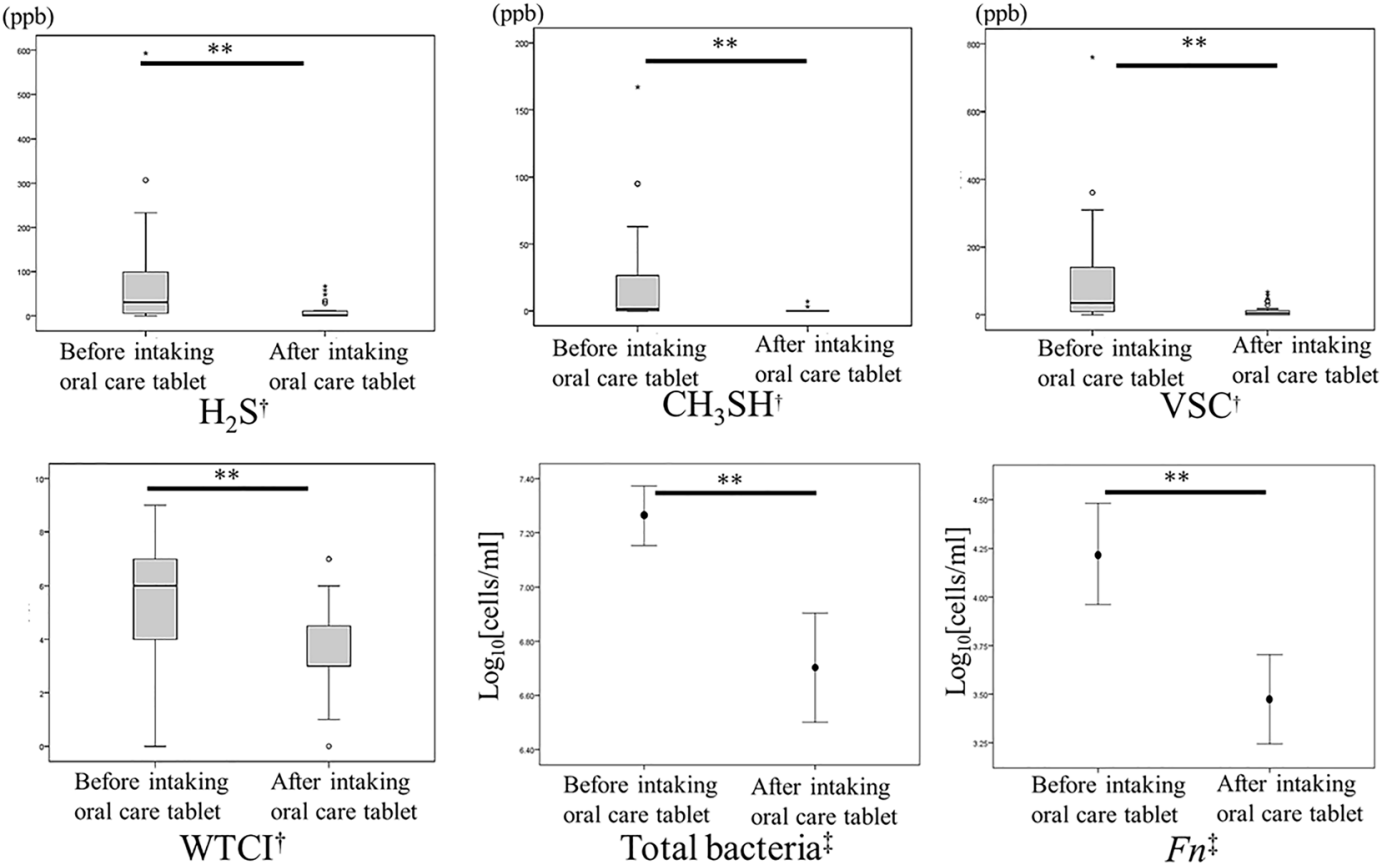
The effect in taking oral care tablet (Intervention II). H_2_S, CH_3_SH, volatile sulfur compound (VSC), and Winkel tongue‐coating index (WTCI) were presented medians with interquartile range, and both bacterial counts were presented as mean with 95% confidence interval. ^†^Wilcoxon signed‐ranks test.^‡^Paired *t* test.^*^ *p* < .05,^**^ *p* < .01

There were significant differences in terms of the level of H_2_S, VSC, and WTCI at Intervention I and all evaluation values at Intervention II, as shown in Figure 4. Regarding Intervention III, the final number of subjects were 30 because two students dropped out. There were significant differences in terms of the level of H_2_S, VSC, WTCI, the number of total bacteria, and *F. nucleatum* at Intervention III, as shown in Table 4.

**Table 4:** Comparison of the effect in each intervention study

	Intervention I	Intervention II	Intervention III
H_2_S^a^	*p* = .031*	*p* < .01**	*p* < .01**
CH_3_SH^a^	*p* = .246	*p* < .01**	*p*=0.192
VSC^a^	*p* = .021*	*p* < .01**	*p* < .01**
WTCI^a^	*p* < .01**	*p* < .01**	*p* < .01**
Total bacteria^b^	*p* = .327	*p* < .01**	*p* < .01**
*Fn* b	*p* = .923	*p* < .01**	*p* < .01**

Abbreviations: VSC, volatile sulfur compound; WTCI, Winkel tongue‐coating index.

a Mann–Whitney *U* test.

b Student's *t* test.

* *p* < .05,

** *p* < .01.

### 3.3 Comparison of Interventions I and II or Interventions I and III

**Figure 5:**
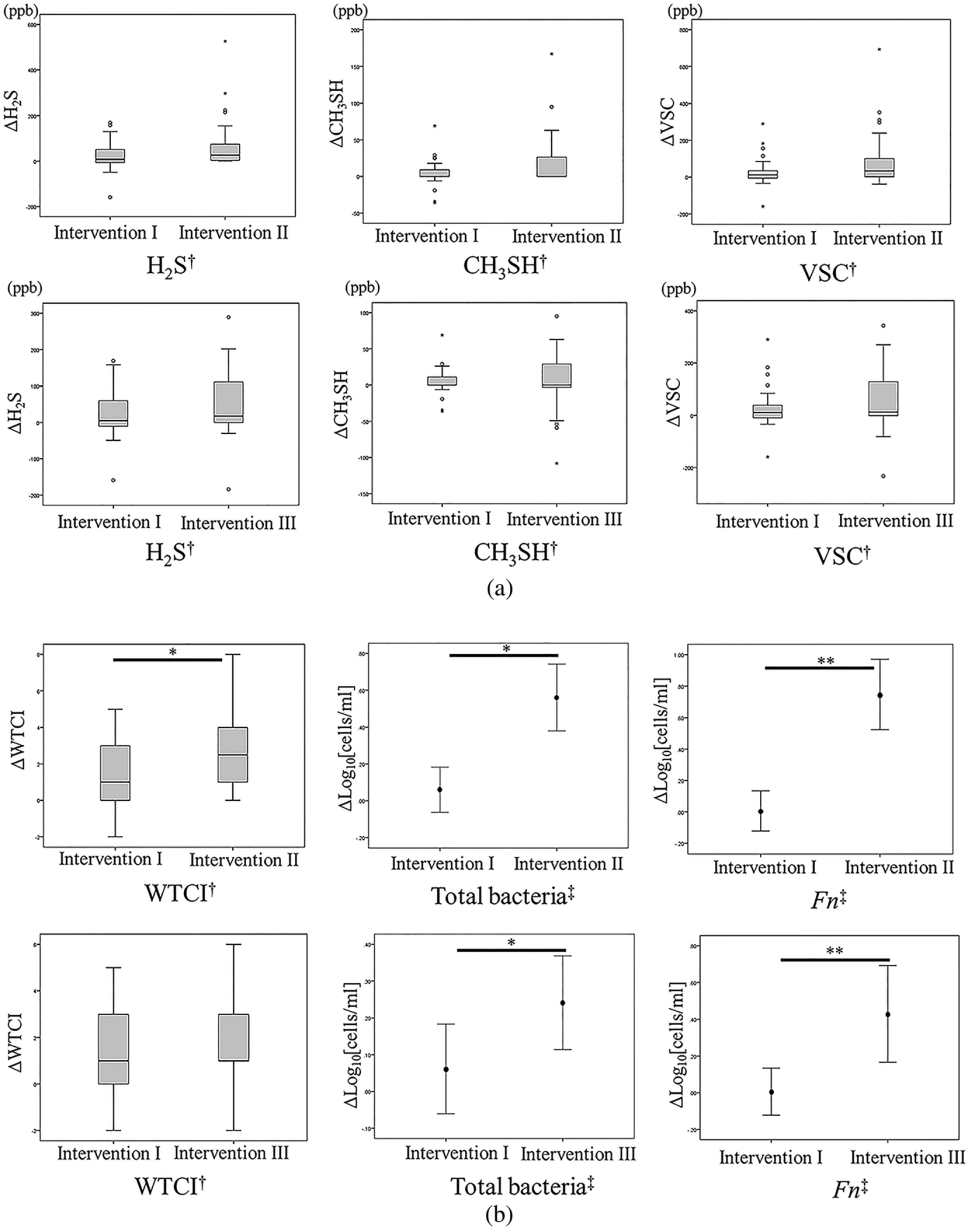
Comparison of the methods between Interventions I and II or Interventions I and III. 5a and 5b showed the results of parameters regarding oral malodor and tongue coating bacteria, respectively. H_2_S, CH_3_SH, volatile sulfur compound (VSC), and Winkel tongue‐coating index (WTCI) were presented medians with interquartile range, and both bacterial counts were presented as mean with 95% confidence interval. ^†^Mann–Whitney *U* test.^‡^Student's *t* test.^*^ *p* < .05,^**^ *p* < .01

There was no significant difference observed in the items of oral malodor (Figure 5a). By the comparison of Interventions I and II, it revealed by the analysis of this crossover study that the value of WTCI decreased significantly after taking tablets than after tongue brushing (Figure 5b). When compared with Interventions I and III, there was no significant difference in this item. A clear difference in the results between Interventions II and III compared with Intervention I was observed in WTCI.

On the other hand, the number of total bacteria and *F. nucleatum* decreased significantly after taking tablets in both intervention (II and III) than after tongue brushing (Figure 5b).

## 4 DISCUSSION

As a result of this clinical trial in healthy, young adults, VSC, WTCI, and the number of total bacteria were effectively reduced by taking oral care tablets containing kiwifruit powder. Moreover, it was newly clarified that even the concentration of *F. nucleatum* in tongue coating was reduced. Generally, it is known that the tongue brush is physically able to remove the tongue coating.(Slot et al., 2015; Yaegaki et al., 2002) Interestingly enough, this study suggested that the oral care tablet was able to suppress not only the tongue coating but also the concentration of *F. nucleatum* in the tongue coating.

Approximately 60% of halitosis cases are reported to be associated with tongue coating.(Yaegaki & Sanada, 1992b) Further, oral periodontopathogenic bacteria can be aspirated into the lung to cause aspiration pneumonia in older adults and individuals with a weakened immune system.(Terpenning, 2005) Therefore, the removal of tongue coating, which leads to the reduction of oral bacteria, is important in maintaining oral and systemic health. Notably, we have focused on *F. nucleatum* among oral bacteria. *F. nucleatum*, a gram‐negative anaerobic oral bacterium, produces large amounts of VSCs including H_2_S and CH_3_SH (Claesson et al., 1990) and is a representative for the occurrence of oral malodor. These bacteria are frequently isolated from tongue coating regardless of periodontal condition.(Chew, Zilm, Fuss, & Gully, 2012; Signat, Roques, Poulet, & Duffaut, 2011) In addition, it was reported that this is used as a landmark for the effect of tongue cleaning in clinical studies.(Matsui et al., 2002) Moreover, it has been proposed that *F. nucleatum* binds to early colonizers and acts as a bridging organism that mediates coadherence of disease‐causing late colonizers such as *Porphyromonas gingivalis* to dental biofilms.(Kolenbrander et al., 2002) Therefore, *F. nucleatum* plays a central role in bacterial aggregation, biofilm maturation, and pathogenicity of oral biofilm in the oral cavity. In other words, the removal of *F. nucleatum* in the tongue coating is essential to prevent oral health problems. On the other hand, our previous study showed that (a) 70% of patients who highly accumulated tongue coating did not recognize their tongue coating and (b) half of them had no habit of daily tongue cleaning.(Amou et al., 2014) Therefore, simple and easy‐to‐continue tongue care is considered to be very useful for individuals with accumulated tongue coating.

Generally, a tongue brush has been used for tongue care. A tongue brush is effective in the removal of tongue coating and the halitosis control.(Amou et al., 2014) There are various reports on the reduction of bacterial count on the tongue by tongue brushing.(Gilmore & Bhaskar, 1972; Laleman, Koop, Teughels, Dekeyser, & Quirynen, 2018; Matsui et al., 2002) Bordas et al. (Bordas et al., 2008) reported that although mechanical tongue cleaning without chemical intervention can reduce bacterial load on the tongue, this effect is transient, and regular tongue cleaning is required to provide a long‐lasting reduction in bacterial numbers. However, there are some problems with the tongue brush. Quirynen et al. (Quirynen et al., 2004) reported that there might be a possibility of damaging the mucous membrane of the tongue by the bristle of tongue brush, and there was a possibility of triggering the gag reflex during tongue brushing. Moreover, they described that the complex surface properties of the tongue dorsum might have prevented the bristle of tongue brush from reaching deep into the grooves.

We focused on the oral care tablet because oral care tablets are common, and they can be taken easily. Licking an oral care tablet needs less physical activity than brushing the tongue with a tongue brush. Therefore, the oral care tablet may be able to solve the problems of the tongue brush. Yoshimatsu et al. (Yoshimatsu et al., 2006; Yoshimatsu et al., 2007) showed that oral care tablets were effective in reducing both tongue coating and VSCs. This oral care tablet has a rough surface allowing the easy removal of the tongue coating while licking it, and it also contains cysteine protease actinidin, extracted from kiwifruit. Regarding the mechanism of the removal of tongue coating by oral care tablets, it is considered that tongue coating can be removed with both the chemical degradation by actinidin and the mechanical effect by the rough surface of the tablet as reported previously.(Mugita, Takahashi, & Komasa, 2016; Nohno et al., 2012) There is a difference in composition of the tablet between previous studies (Nohno et al., 2012; Yoshimatsu et al., 2006; Yoshimatsu et al., 2007) and the present study. Compared with the tablet used in the previous study, we have reduced the amount of actinidin and added two types of sugar alcohols to the present tablet for rough surface. The time for disintegration of this tablet was obtained from the preliminary study, the average ± standard deviation was 5.4 ± 1.5 min per tablet. However, typical side effects, such as membrane irritation, were not found in this clinical trial. Thus, we considered that the oral care tablet could be used more efficiently for tongue care than the tongue brush.

In this study, we compared the effects of the oral care tablet and the tongue brush to clarify its potential to be an effective care for tongue coating, oral malodor, and oral bacteria. Significant reductions in WTCI and VSC were confirmed for both taking tablets and tongue brushing as previously reported.(Amou et al., 2014; Yoshimatsu et al., 2006; Yoshimatsu et al., 2007) Although tongue brushing did not show any effects on the number of total bacteria and *F. nucleatum*, taking tablets significantly decreased them. Our previous observation study suggests that tongue cleaning may be an effective method for improving halitosis.(Amou et al., 2014) However, it was revealed in this intervention study that the amount of total bacteria and the bacteria related to halitosis was not reduced by tongue brushing effectively, whereas it was reduced by oral care tablet effectively. This is the first observation in this field. The oral care tablet contained food ingredients such as kiwifruit powder. Also, mechanical removal occurred due to the rough surface of the tablet. This study showed new findings regarding the effect on oral bacteria by the mechanical and chemical action of tablets. These combined factors might contribute significant effects not only on WTCI but also on the concentration of total bacteria and *F. nucleatum. F. nucleatum* plays a role of "bridge" between early and late colonizers and is a key bacterium in biofilm formation on the tongue and tooth surface. According to the result of being able to remove *F. nucleatum* effectively, this oral care tablet might be a useful tool for the removal of oral biofilm. We also obtained a difference of WTCI in the results between Interventions II and III; this is speculated that restoring accumulation of tongue coating occurred overnight. Therefore, it might be preferable to take tablets after tongue brushing.

Analysis of our data showed that the oral care tablet could be used more easily for tongue care than the tongue brush and could be an effective tool for the prevention of oral malodor. Moreover, in terms of the reduction of pathogenicity in the oral biofilm, the oral care tablet could contribute to disease prevention because it also decreased *F. nucleatum* on the tongue. Pneumonia is a major cause of death for the elderly and the care recipient. Among them, aspiration pneumonia, which is caused by bacterial infection resulting from the entrance of foreign materials such as food and saliva into the lung, is a serious problem for the elderly.(Teramoto et al., 2008) For the prevention of aspiration pneumonia, it is important to reduce the number of bacteria in the oral cavity by removing tongue coating and dental plaque. This tablet may increase salivary flow and the risk of swallowing by mistake when taken by elderly people. However, the increase of salivary flow led to the decrease in total bacterial amount. Because this oral care tablet reduced the number of bacteria, including *F. nucleatum* on the tongue in this study, it might also be helpful to prevent aspiration pneumonia.

Furthermore, a recent study showed that *F. nucleatum* had been implicated in colorectal cancer (Brennan & Garrett, 2019) and esophageal cancer.(Yamamura et al., 2016) Further studies will clarify whether the reduction of oral bacteria, including *F. nucleatum* in the oral cavity leads to the prevention of these diseases.

There are several limitations in this study. Our data were obtained only from young adults, this is the limitation of generalizability. As potential bias, the dental plaque accumulation was not monitored whereas it may influence tongue coating bacteria as reported previously.(Matsui et al., 2002) It is possible that the decrease in total bacterial amount is due to an increase in salivary flow stimulated by the ingredients of the tablet. However, we could not measure the alternation of salivary flow rate. We obtained interesting results by analyzing *F. nucleatum* as halitosis‐related bacteria. However, it will be better to add and analyze other halitosis‐related bacteria to confirm the effect of oral care tablet. Further study is needed to confirm these phenomena.

## 5 CONCLUSION

These results suggested that an oral care tablet containing kiwifruit powder might be effective in reducing total bacteria and *F. nucleatum* in tongue coating in addition to VSC, which causes oral malodor.

### CLINICAL RELEVANCE

#### Scientific rationale of the study

Tongue brushing is one method for removing tongue coating. However, some of the problems in self‐care approach still remain. It is, therefore, apparent to establish an effective method for removing tongue coating compared with tongue brushing.

#### Principal findings

The use of oral care tablet containing kiwifruit powder was effective in reducing oral bacteria on tongue dorsum in addition to reducing VSC in breath odor when comparing with tongue brushing.

#### Practical implications

The use of oral care tablet was an effective method in reducing oral bacteria and VSC. Therefore, dental hygienists should be able to recommend oral care tablets containing kiwifruit powder as one of the effective tongue cleaning methods for patients with thick tongue coating.

## FUNDING INFORMATION

Daisuke Hinode received financial support from JSPS KAKENHI Grant 16K11860 from the Japan Society for the Promotion of Science. Hiroki Asakuma and Hiroshi Takii are employees of Ezaki Glico Co., Ltd. and part of this study was funded by Ezaki Glico Co., Ltd.

## CONFLICT OF INTEREST

Yuki Matsumura, Makoto Fukui, and Masami Yoshioka report no conflict of interests.

## AUTHOR CONTRIBUTIONS

The authors' contributions are as follows: Yuki Matsumura performed all experiments and drafted the paper. Daisuke Hinode designed and coordinated the study and drafted the paper. Makoto Fukui and Masami Yoshioka obtained the clinical data from subjects and performed some of the experiments. Hiroki Asakuma and Hiroshi Takii contributed in designing and drafting the paper. All authors reviewed the paper critically for content and approved it for submission.
